# Counting Canola: Toward Generalizable Aerial Plant Detection Models

**DOI:** 10.34133/plantphenomics.0268

**Published:** 2024-11-08

**Authors:** Erik Andvaag, Kaylie Krys, Steven J. Shirtliffe, Ian Stavness

**Affiliations:** ^1^ Department of Computer Science, University of Saskatchewan, Saskatoon, Canada.; ^2^ Department of Plant Sciences, University of Saskatchewan, Saskatoon, Canada.

## Abstract

Plant population counts are highly valued by crop producers as important early-season indicators of field health. Traditionally, emergence rate estimates have been acquired through manual counting, an approach that is labor-intensive and relies heavily on sampling techniques. By applying deep learning-based object detection models to aerial field imagery, accurate plant population counts can be obtained for much larger areas of a field. Unfortunately, current detection models often perform poorly when they are faced with image conditions that do not closely resemble the data found in their training sets. In this paper, we explore how specific facets of a plant detector’s training set can affect its ability to generalize to unseen image sets. In particular, we examine how a plant detection model’s generalizability is influenced by the size, diversity, and quality of its training data. Our experiments show that the gap between in-distribution and out-of-distribution performance cannot be closed by merely increasing the size of a model’s training set. We also demonstrate the importance of training set diversity in producing generalizable models, and show how different types of annotation noise can elicit different model behaviors in out-of-distribution test sets. We conduct our investigations with a large and diverse dataset of canola field imagery that we assembled over several years. We also present a new web tool, Canola Counter, which is specifically designed for remote-sensed aerial plant detection tasks. We use the Canola Counter tool to prepare our annotated canola seedling dataset and conduct our experiments. Both our dataset and web tool are publicly available.

## Introduction

Early-season plant population estimates are highly valued by canola researchers and producers as important indicators of crop health. Low-density plant stands often struggle to compete with weeds and suffer greater soil moisture loss [1]. Sparse stands are also more susceptible to insect and disease pressure [2]. As a result, poor emergence is often associated with reduced yield [3]. Emergence rate information can help inform management practices, which can, in turn, lead to better returns at harvesttime.

Traditionally, plant emergence rates have been estimated with manual counting methods: small areas of a field are randomly chosen and the seedlings within these areas are counted. Due to the large amount of labor involved, this approach relies heavily on sampling techniques and can only provide a very fragmentary view of emergence rates across a field.

In recent years, a new approach to plant counting has been proposed. First, an unmanned aerial vehicle (UAV) is used to acquire aerial images of a field. Following this, a deep learning-based object detection model is applied to the imagery, counting the individual seedlings in each image. Compared to manual methods, this new approach to counting can potentially deliver population estimates for much larger areas of a field, providing canola producers with a better understanding of the variation in plant density throughout their fields.

Previous studies have convincingly shown that deep learning-based models can accurately detect plants and plant organs in aerial imagery. Corn plants [4], wheat spikes [5], sorghum panicles [6], rice seedlings [7], potato plants [8], and many other kinds of commercial crops have been successfully detected from aerial images.

A few studies have examined the task of aerial canola seedling detection in particular. Madsen et al. [9] showed that seedlings from a variety of plant species, including canola, could be identified in overhead images. Zhang et al. [10] and Zhao et al. [11] estimated canola seedling population counts from low-resolution drone images. Higgs et al. [12] used a Faster R-CNN (region-based convolutional neural network) model [13] to detect canola seedlings in images acquired from a custom tractor-based platform.

Although these studies have demonstrated the viability of canola seedling detection, each one has been limited to a relatively narrow set of field conditions. Unfortunately, developing detection models that generalize well across a variety of locations and conditions is a major challenge. Existing research has shown that the performance of deep learning-based models often degrades in the presence of distribution shift; that is, when the set of image conditions encountered during deployment differs from the set of conditions found within the model’s training set [14].

Distribution shift is an extremely common phenomenon in agricultural detection settings due to the wide variety of factors that can influence an image’s appearance. Weather and lighting effects, capture settings, and various field conditions can all profoundly affect the appearance of plant objects within an image.

Canola imagery in particular can vary considerably in appearance due to the wide range of environments in which canola is grown. Since its introduction in the 1970s, canola production within the Canadian prairie provinces has steadily expanded in area, and now encompasses a range of climate and soil zones [15]. Varieties of canola are also grown all over the world; besides Canada, major producers include China, Australia, and Europe [16]. As a result, canola fields can vary dramatically in appearance, depending on a range of site-specific factors.

Developing detection models that can generalize to a variety of image domains is therefore of great importance in the development of practical canola counting systems. Three aspects of a model’s training data have been identified as critical factors in improving generalizability: the size of the training set, the diversity of the training data, and the quality of the object annotations [14].

In this paper, we seek to better understand the effects of these 3 factors within the context of aerial canola seedling detection. In particular, we focus on how these factors influence a model’s ability to perform on in-distribution (ID) images (images that are drawn from the same acquisition sessions as the training data) and out-of-distribution (OOD) images (images that were acquired at different times and locations than those found in the model’s training set).

The main contributions of our paper include the following:

• We conduct a number of experiments that investigate how different training set attributes (size, diversity, and quality) affect a plant detection model’s ability to generalize to unseen image sets.

• We present a large, diverse, and high-quality dataset of aerial canola field imagery, collected across multiple locations over several years. We make this dataset publicly available.

• We present an open source web tool, Canola Counter, that can be used to perform remote-sensed aerial plant detection tasks.

## Materials and Methods

### The Canola Counter web tool

Aerial plant detection is fundamentally a cross-disciplinary task, requiring the expertise of both crop scientists and computer vision researchers. In order to facilitate collaborative work on this task, we developed a software tool called Canola Counter, which we used extensively in this study.

Canola Counter is a web-based platform for performing agriculture-based object detection tasks, with a special emphasis on aerial plant detection. The tool allows users to upload sets of images, view and annotate image data, and train, fine-tune, and apply object detection models. Canola Counter contains a number of features that are particularly designed to support aerial plant phenotyping tasks, including vegetation segmentation tools, support for orthomosaic images, and geospatial visualizations. The tool also provides outputs that are of particular interest to crop researchers studying emergence rates, such as object counts for user-defined regions, object density estimates (counts per square metre), and measurements of plant size and spacing.

**Fig. 1.**
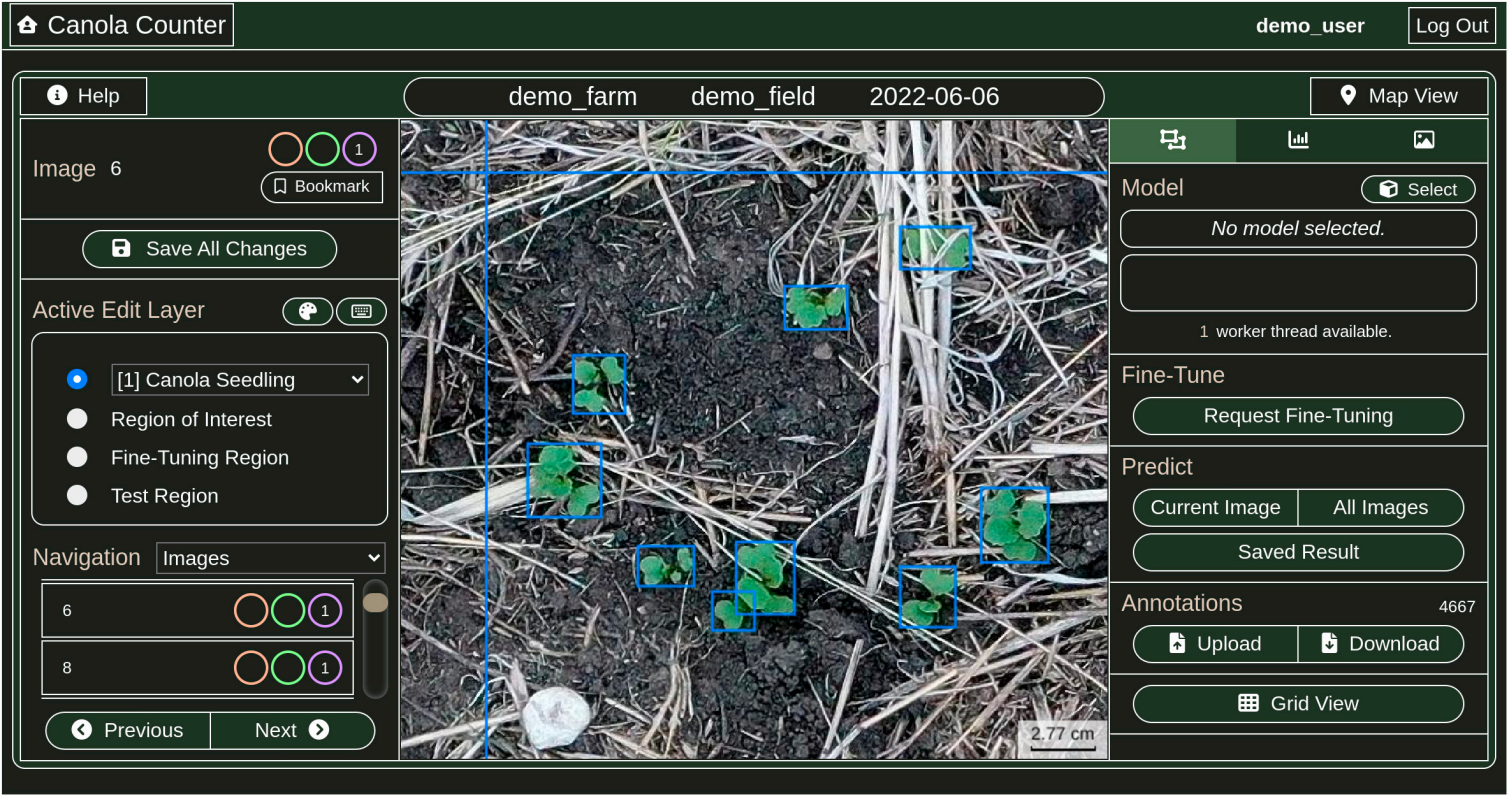
Screenshot of the Canola Counter web tool, which we used to prepare our annotated canola seedling dataset and run our experiments.

A screenshot of the Canola Counter workspace is shown in Fig. 1. We used the Canola Counter tool to collaboratively annotate a large number of canola seedling image sets (“Dataset preparation” section) and run the experiments described in the “Experimental design” section. Additional information about the Canola Counter tool can be found in [17].

### Dataset preparation

In order to examine the effects of training set size, diversity, and quality within an aerial plant detection context, we assembled a large dataset of early-season canola field images. We acquired these images over the 2021, 2022, and 2023 growing seasons from a variety of small-plot research fields and production fields within the province of Saskatchewan, Canada. All of our imagery was acquired with a Hasselblad L1D-20c camera mounted on a DJI Mavic 2 Pro Drone, which we flew at a height of 2 m.

Canola plants first emerge by pushing a pair of leaf-like organs (called cotyledons) out of the soil [18]. As the seedling continues to grow, true leaves will begin to develop from the center of the plant. The plants in our images are primarily at the cotyledon stage of development, since it is at this growth stage that individual plant identification is easiest. Our dataset also includes many examples of canola plants with small numbers of true leaves—generally 4 or fewer leaves. We found that individual seedlings could not be reliably identified in most images captured after the 4-leaf stage, due to the degree of overlap between neighbouring plants.

We used the Canola Counter tool to annotate our canola seedling imagery. All box annotations were created by hand, without model assistance. The annotators identified canola seedlings by looking for the characteristic shape of opposing cotyledon pairs, allowing for the possibility of true leaves in the centers of the seedlings. Each bounding box in our dataset tightly encloses a single canola seedling object. We allowed the bounding boxes to overlap with each other when the seedlings overlapped.

We define an image set as a collection of images obtained during a single acquisition session (drone flight) on a single field. In total, our annotated dataset consists of 54 image sets. We used 27 of these image sets for training and ID testing, and we used the remaining 27 image sets for OOD testing (Table 1). Each of our image sets has a characteristic appearance that arises from a unique combination of various field-based and capture-based conditions, including the growth stage of the seedlings, the uniformity of plant spacing, the degree of occlusion from stubble, the lighting and weather conditions at the time the field was imaged, and the quantity and species of weeds found in the field. Therefore, we consider each image set to represent a separate “domain”. This follows the approach used by David et al. [14]. We consider a canola seedling model to generalize well if it can produce strong performance on image sets (domains) that are unseen during model training.

**Table 1.** Summary annotation statistics for our canola seedling dataset

	No. of image sets	No. of images	No. of annotations
Train	27	323	57,237
ID test	27	54	9,686
OOD test	27	54	10,136

**Fig. 2.**
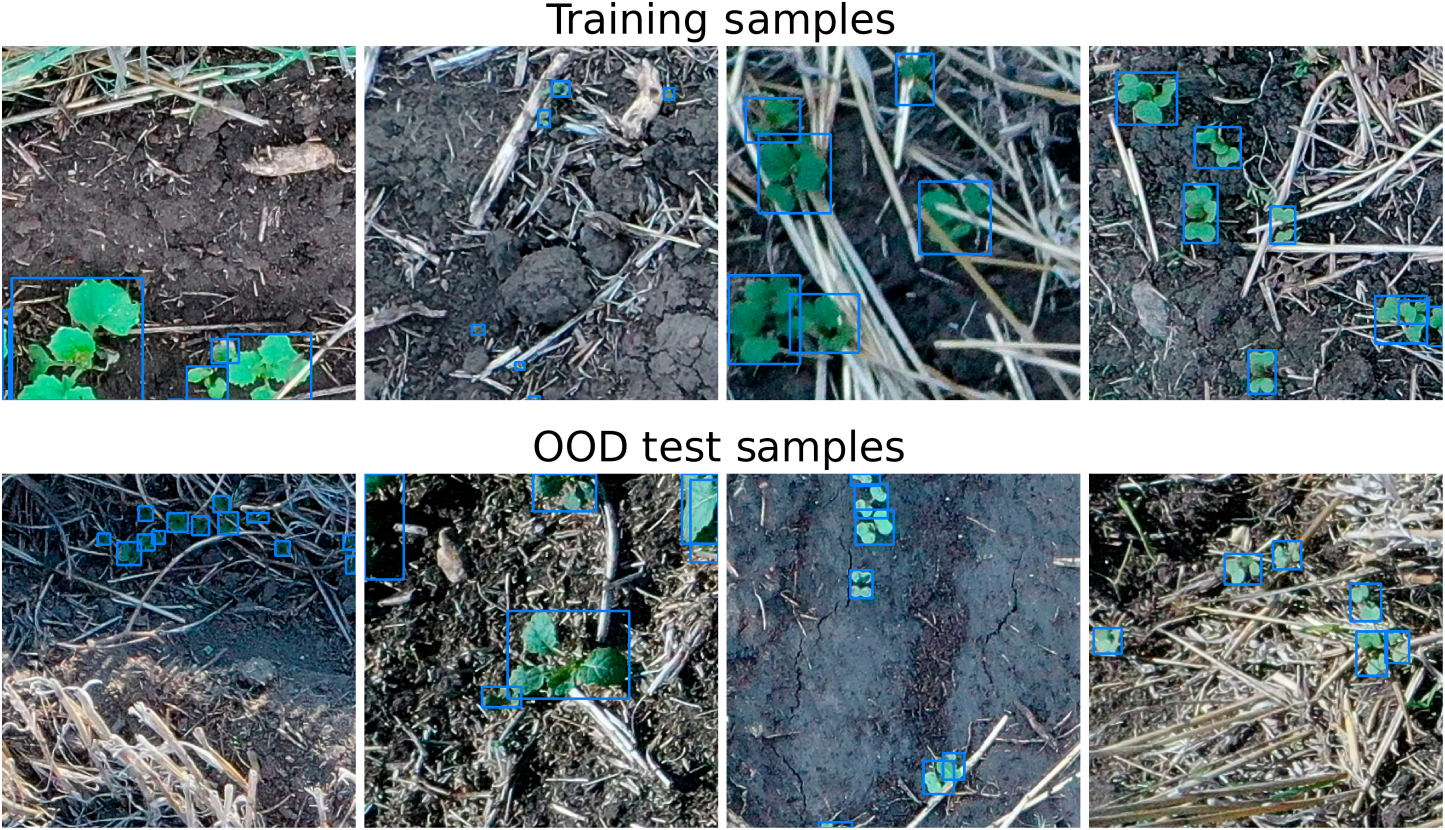
Sample 416 × 416 image patches extracted from 4 of our training sets and 4 of our OOD test sets.

Figure 2 shows representative image patches from 8 of our canola seedling image sets. The figure illustrates the diverse visual conditions found within our dataset. Among other factors, substantial variation in object size, image sharpness, occlusion, and lighting conditions can be readily observed.

### Experimental design

We used our canola seedling dataset to conduct a series of experiments that explore the ways in which a model’s OOD performance is affected by specific aspects of its training set. All of these experiments were conducted with the YOLOv4 Tiny model architecture [19], which we chose for its speed and performance. All models were trained for 200 epochs. We used the same multipart loss function proposed by the original authors of YOLOv4, which consists of a complete intersection-over-union (IoU) box regression loss term [20], a classification loss term (categorical crossentropy), and a confidence loss term (categorical crossentropy with focal modulation [21]). After training, the set of weights that produced the lowest training loss was selected. Models were trained with an Adam optimizer [22] with a batch size of 16 and an initial learning rate of 1e−4. During training, we made use of a small set of data augmentation techniques: 90° rotations and horizontal and vertical flipping. These 3 transformations were applied in succession on a per-image basis, each with probability *P* = 0.5.

We divided our images into small patches before training and prediction, since the raw images were too large to be fed directly to the YOLOv4 Tiny detection model. We used the default YOLOv4 Tiny input size of 416 × 416. During training, non-overlapping image patches were extracted. During inference, we extracted patches that overlapped by 50%. We combined patch predictions by discarding the predicted boxes with centers falling inside overlapping areas and by applying non-maximum suppression with an IoU threshold of 0.4.

We chose the accuracy metric from the Global Wheat Head Dataset (GWHD) challenge as our primary performance metric [23]:

$$\text{Accuracy}=\frac{\mathrm{TP}}{\mathrm{TP}+\mathrm{FN}+\mathrm{FP}},$$ (1)

where *TP*, *FN*, and *FP* are the number of true positives (annotated boxes that can be matched with a predicted box), false negatives (annotated boxes that cannot be matched with a predicted box), and false positives (predicted boxes that cannot be matched with an annotated box) within a test image. We use the same thresholds (IoU ≥ 0.5, confidence > 0.5) as the GWHD challenge when determining the number of true positives, false positives, and false negatives in an image. Image set accuracy is measured by averaging the accuracy scores obtained on each image in the set. ID test accuracy is obtained by averaging the accuracy scores of the ID test image sets. OOD test accuracy is obtained analogously.

#### Training set size

In our first experiment, we assessed how training set size affects a plant detection model’s ability to generalize to new image sets. Specifically, we trained detection models on training sets of various sizes and assessed their performance across our ID and OOD test sets. A training set of a given size was assembled by randomly sampling the appropriate number of image patches from across our 27 training sets without replacement. We used the following training set sizes (number of training patches) in this experiment: 250, 500, 1,000, 2,000, 4,000, 8,000, 16,000, 24,000, 32,000, and 38,891. The total number of annotated training patches we have prepared is 38,891. For each training set size, we trained 5 model replications. We report average model performance across these replications.

#### Training set diversity

In our second experiment, we examine the relationship between training set diversity and model generalizability. In particular, we compare the OOD performance of models that have been trained on datasets of equal size, but varying levels of internal diversity.

We conducted our experiment in the following manner. For each of our 27 training image sets, we trained 5 detection models. Each of these models was trained on data drawn from a single image set; we refer to these models as non-diverse models. Following this, we trained 5 detection models on data drawn from across all 27 of our training sets. We refer to these models as diverse models.

We trained every model in this experiment on the same amount of image data: 630 image patches. This allows us to examine the effects of training set diversity independently from the effects of training set size. We chose a training set size of 630 image patches since this is the total number of patches in our smallest training image set. If an image set contained more than 630 annotated image patches, we assembled a training set by sampling from the pool of available image patches without replacement. We used the same procedure when assembling the training sets of the diverse models: all of the available training patches from the 27 training sets were pooled together and 630 patches were randomly sampled without replacement from the pool. We assessed the performance of our diverse and non-diverse models on our 27 OOD test images sets.

#### Training set quality

Our third and final experiment examines the effect of annotation noise on OOD test accuracy. We explore the effects of 2 types of annotation noise: loosely drawn bounding box annotations and missing box annotations.

In order to model loosely drawn bounding boxes, we artificially dilated the box annotations in our 27 training sets by applying the following transformation:

$$\begin{array}{c} \text{min}\;\_\;x=\text{min}\;\_\;x-U\left\{0,N\right\}\\ \text{min}\;\_\;y=\text{min}\;\_\;y-U\left\{0,N\right\}\\ \text{max}\;\_\;x=\text{max}\;\_\;x+U\left\{0,N\right\}\\ \text{max}\;\_\;y=\text{max}\;\_\;y+U\left\{0,N\right\} \end{array}$$ (2)

where min_x, min_y, max_x, and max_y are the bounding box’s coordinates (in pixel values), $U\in \left[0,N\right]$ is the discrete uniform distribution, and *N* is the maximum amount the bounding box can be expanded in each direction (in pixels). We explored different levels of noise severity by varying the value of *N* from 0 to 32 (inclusive), using a step size of 2.

In order to model missing box annotations, we randomly removed boxes from our training image sets with probability *P*. We used the following values of *P*: 0, 0.05, 0.1, 0.15, 0.25, 0.5, 0.75, and 0.9.

For both noise types, we trained 5 model replications at each noise level. The training sets in this experiment each contained 16,000 image patches, which were randomly sampled (without replacement) from our 27 image sets after the noise effect was applied.

For this experiment, we used 2 metrics to assess model performance on our OOD test sets. In addition to the accuracy metric used in the preceding experiments, we also measured the average absolute difference between the annotated object counts and predicted object counts across our OOD test images. We include this metric for its relevance to the task of aerial plant counting. In many cases, accurate plant population counts are of more interest to crop producers than precise plant locations.

## Results

### Training set size

**Fig. 3.**
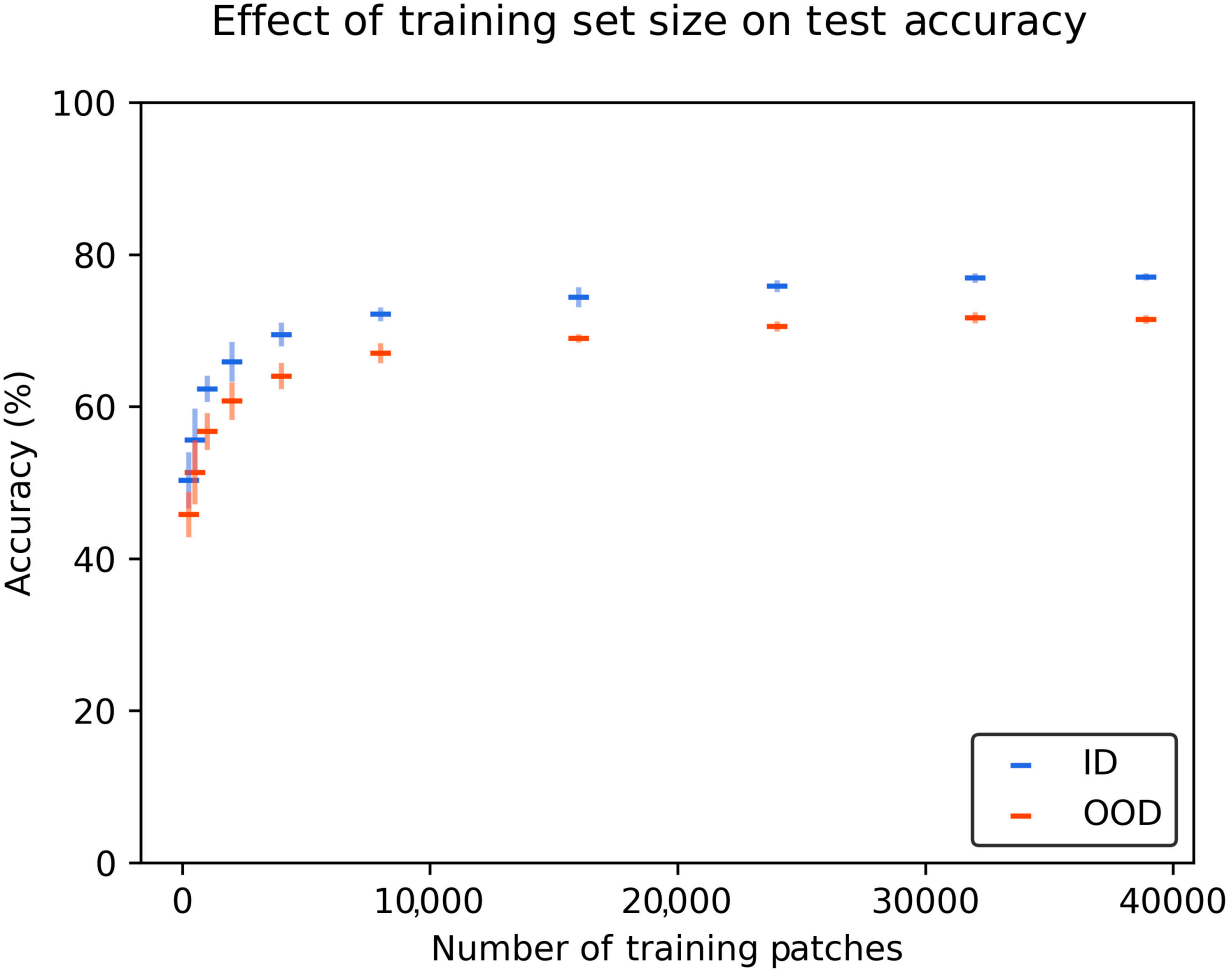
Detection accuracy vs. training set size for ID (blue) and OOD (red) test sets. Horizontal bars indicate mean performance across 5 model replications. Vertical bars indicate the standard error of the mean.

We observe a logarithmic relationship between training set size and model performance (Fig. 3). This relationship holds for both our ID and OOD test sets. We also note that performance consistency across training replications improves as the size of the training set increases. It is reasonable that models trained on larger datasets will tend to perform more consistently across diverse test conditions, as there is a lower chance that the training process will lead to sets of weights that behave erratically in certain parts of the input space. However, our experimental design may also intensify the effect, as our larger training sets have more shared data between them.

Across all training set sizes, ID accuracy exceeds OOD accuracy by an average of 5.16 percentage points (*μ* = 5.16, *σ* = 0.43). The consistency of the ID–OOD gap across training set sizes is notable, and perhaps surprising. It suggests that our training data are, in fact, quite relevant to our OOD test data, as our models’ OOD accuracy improves in lockstep with their ID accuracy, even at large training set sizes. At the largest training set sizes we tested, both ID and OOD accuracy appear to plateau. It seems unlikely that the ID–OOD gap can ever be closed by simply training on additional data drawn from our current training distribution.

Following David et al. [14], we created a mixed-to-test dataset split to assess if the ID–OOD gap could be reduced by training on data from our OOD image sets. To create this new dataset split, we removed half of our OOD test data (one image from each of the 27 OOD test sets) and used these data to replace samples from our training dataset (one image from each of the 27 training image sets). In this way, we changed the composition of our training set, but preserved its size.

We trained 5 detection models on our mixed training set and assessed their performance on our ID test sets and the remaining data from our OOD test sets. We then evaluated the performance of the models from our original dataset split (the models trained on all 38,891 training patches) on these 2 test sets (ID and subsampled OOD).

The results suggest that distribution shift is at least partially responsible for the ID–OOD performance gap seen in our initial experiment (Table 2). Training on samples from the OOD image sets did slightly improve model performance on the remaining OOD data. At the same time, performance on the ID test sets was not negatively affected by the substitution of some of the ID training data for OOD data.

**Table 2.** Mixed-to-test performance comparison. Parentheses show standard deviation across 5 replications.

Training set composition	ID test accuracy (%)	OOD test accuracy (%)
Original	77.1 (0.1)	71.9 (0.3)
Mixed-to-test	77.1 (0.5)	73.3 (0.6)

Nevertheless, the improvement provided by the mixed-to-test training set is quite modest, and a substantial ID–OOD gap remains. It may be the case that the ID–OOD gap could be further closed by training on additional data from our OOD test sets. The small sizes of our OOD test sets prevented us from testing this hypothesis, since we needed to retain some data for testing.

### Training set diversity

**Fig. 4.**
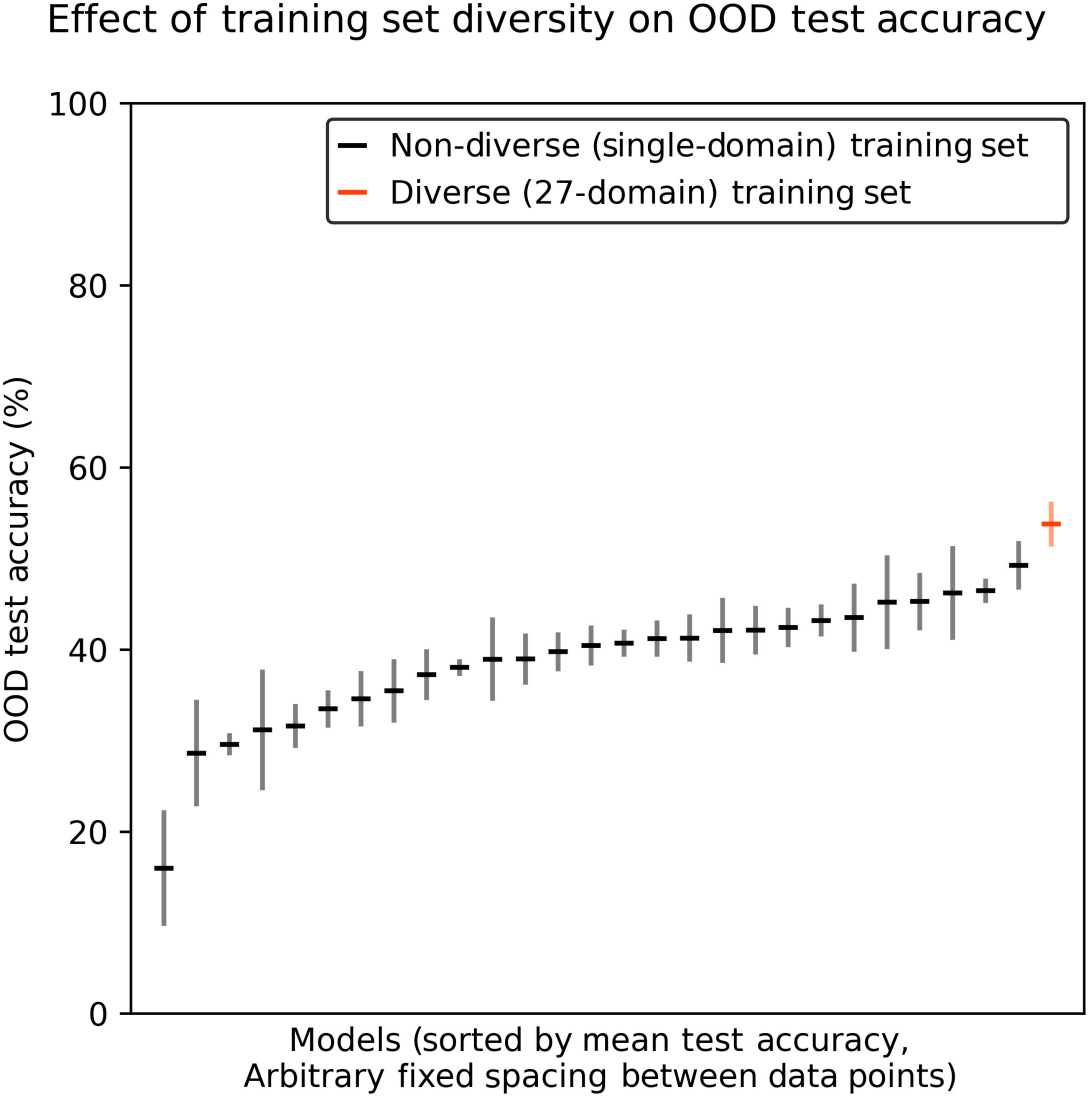
OOD test accuracy of models with diverse (red) and non-diverse (black) training sets. Horizontal bars indicate mean performance across 5 model replications. Vertical bars indicate the standard error of the mean.

Our second experiment shows that diverse training data improve a model’s ability to generalize well to a broad set of unseen image conditions. As seen in Fig. 4, the average OOD test accuracy of our models with diverse training data exceeded the average accuracy of any of our models with non-diverse training sets.

The large variation in performance among our various non-diverse models is also quite striking and suggests that there are substantial differences in the internal diversity of our 27 training image sets. Performance consistency across replications is also extremely variable between the various models in this experiment, and not clearly related to mean accuracy. Notably, our diverse model is quite consistent across replications.

It is fairly unsurprising that training set diversity generally improves a model’s ability to perform well across a wide variety of test images. However, if a model only needs to function well across a narrow set of test conditions, it is less clear if diversity is always beneficial. Training data relevance is also an important factor to consider.

In order to investigate the trade-off between training data diversity and relevance, we assessed how the results of our experiment would change if we evaluated our models on a smaller number of OOD test sets. For each value of *n* between 1 and 27, we evaluated the models from this experiment on 1,000 random samples of *n* test image sets. For each sample of test sets, we determined the difference in accuracy between the diverse model and the best-performing non-diverse model. We then calculated the mean difference in accuracy for each value of *n*.

**Fig. 5.**
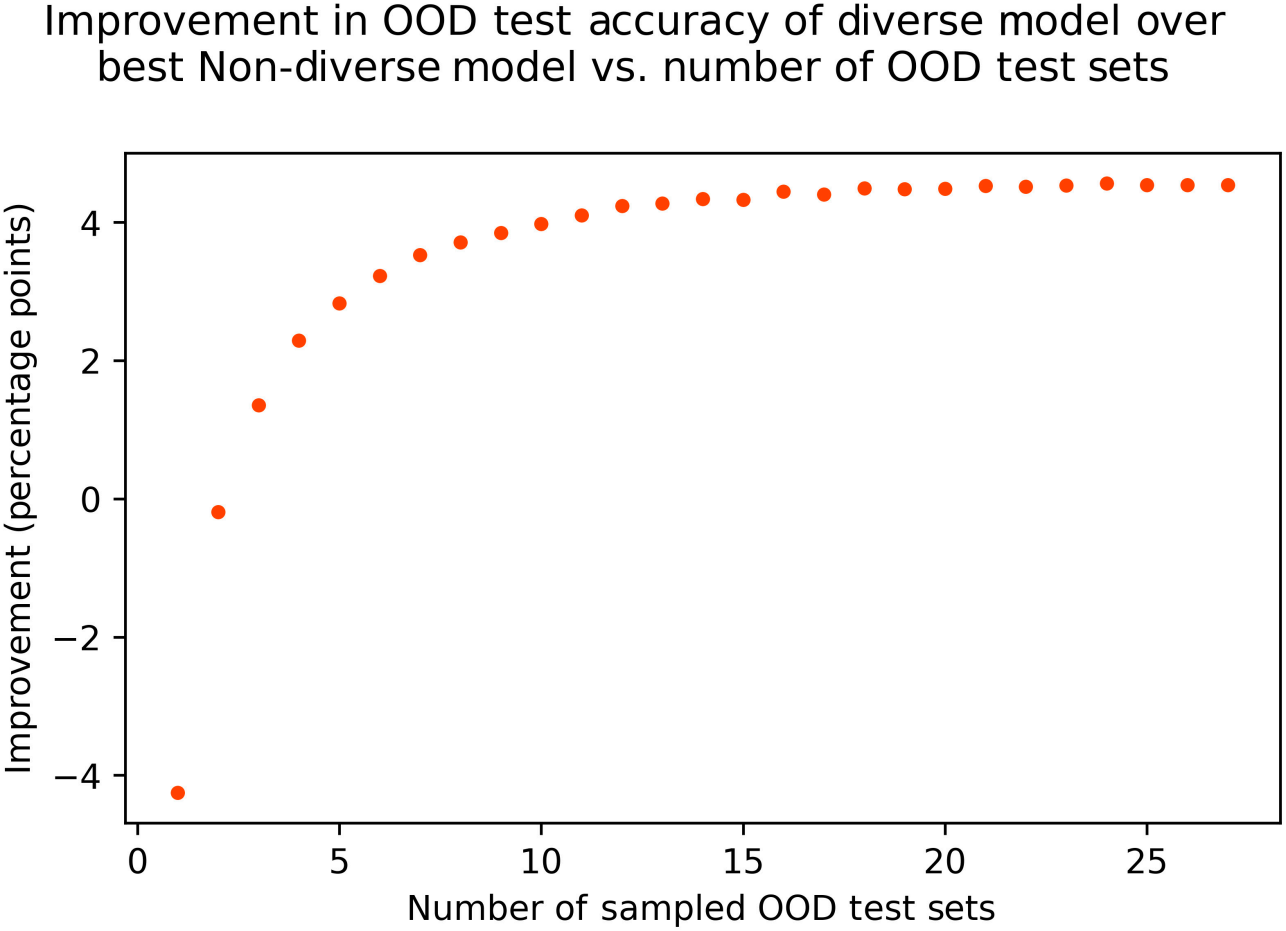
As the number of image sets used for OOD evaluation increases, models with a diverse training set quickly outperform models with a non-diverse training set.

Figure 5 shows the results of this analysis. Interestingly, when a single image set is used to measure OOD test performance, the accuracy of the diverse model is, on average, about 4 percentage points lower than the best-performing non-diverse model. This result highlights the importance of data relevance. At the same time, it is clear that a diverse training set is advantageous for models that need to perform well across multiple domains. It is also noteworthy that the performance advantage of our diverse model appears to plateau as the number of test sets continues to increase. This suggests that the diversity contained within our 27 OOD test sets can generally be captured by fewer than 27 test sets.

### Training set quality

**Fig. 6.**
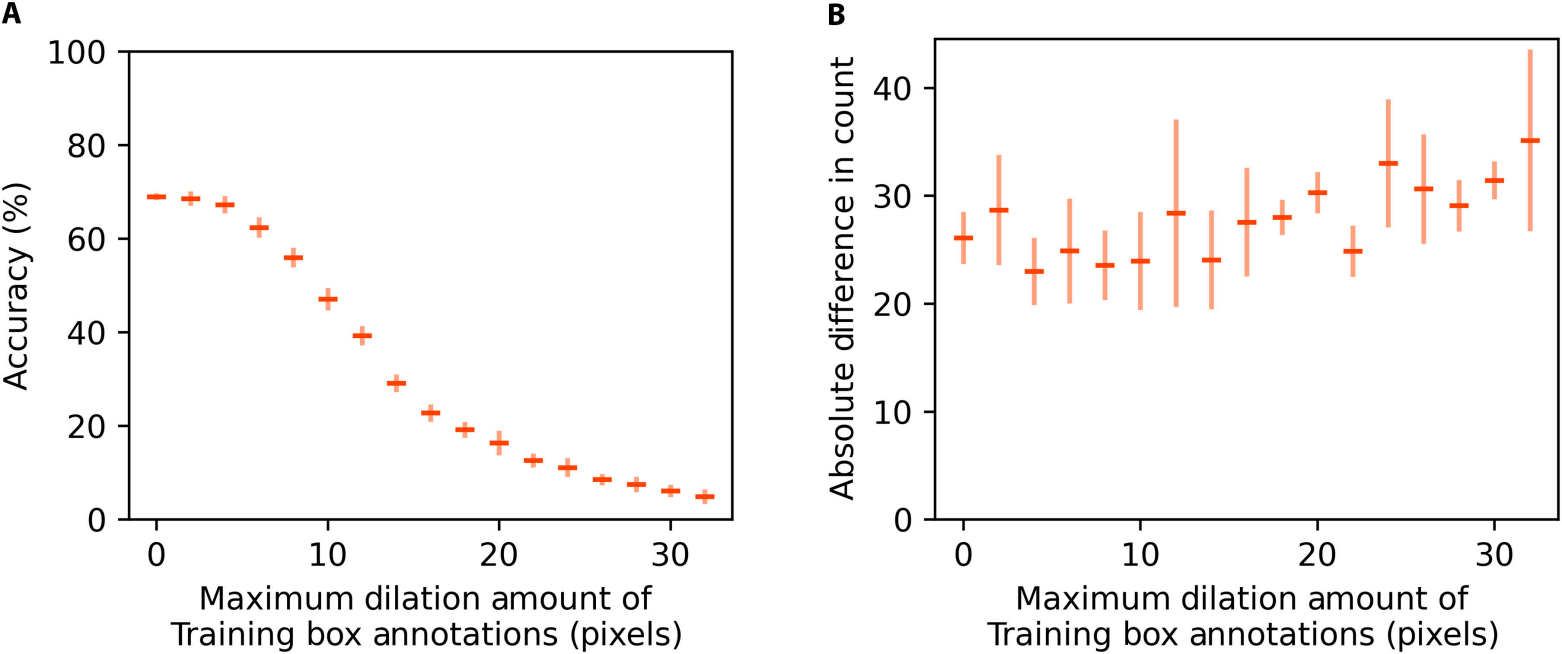
Detection accuracy (A) and count accuracy (B) on OOD test sets vs. level of dilation noise in training data. Horizontal bars indicate mean performance across 5 model replications. Vertical bars indicate the standard error of the mean.

**Fig. 7.**
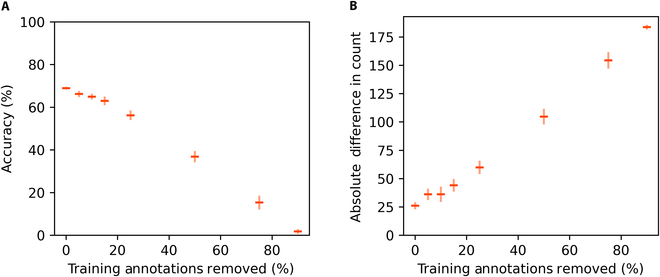
Detection accuracy (A) and count accuracy (B) on OOD test sets vs. percentage of boxes removed from training data. Horizontal bars indicate mean performance across 5 model replications. Vertical bars indicate the standard error of the mean.

The results of our training set quality experiment are shown in Fig. 6 (effect of annotation dilation) and Fig. 7 (effect of annotation removal). As expected, for both types of noise, higher amounts of noise resulted in larger reductions in test accuracy. However, we discovered that the reason for this decline was different for the 2 types of noise.

Training on dilated bounding box annotations resulted in models that generated dilated box predictions. Although these models were still able to detect the objects in the test images, they were unable to precisely localize the objects. At low levels of dilation noise, the models’ predictions still overlapped substantially with the annotated test boxes. However, the smallest test objects could not be localized at the required IoU threshold of 0.5. As the dilation level increased, objects of ever larger sizes could no longer be localized at the required IoU threshold. Assuming a normal distribution of box sizes within our test sets, the s-shaped curve of accuracy decline that we observe is quite reasonable: the decline in accuracy is steepest when boxes of average size can no longer be localized with sufficient accuracy.

In contrast, training on data with missing annotations produced models that struggled with detection rather than localization. The decline in accuracy of these models is roughly proportional to the fraction of annotations that were removed from their training sets.

Due to these contrasting behaviors, the 2 types of noise have very different effects on a model’s ability to produce accurate object counts. Models trained on loose bounding boxes can still generate fairly accurate object counts, even at high levels of noise. In contrast, training on images with missing annotations will quickly destroy a model’s ability to generate precise object counts.

## Discussion

### Relation to previous work

#### Effect of training set size

In our training set size experiment, we witnessed a logarithmic relationship between training set size and ID model performance. Previous studies using benchmark datasets have also observed this relationship [24,25]. Various plant and fruit detection studies have reported similar findings as well [26–29]. However, the results of these latter studies are sometimes noisier, owing to the smaller sizes of the datasets.

We also observed a logarithmic relationship between training set size and OOD model performance. The effect of training set size on OOD performance has not been studied extensively; however, in a sugar beet and potato plant detection study, Ruigrok et al. [8] also report logarithmic relationships, provided that the training data are relevant to the OOD test data.

In contrast to preceding plant detection studies, our experiment evaluated both ID and OOD performance across multiple training set sizes. We were therefore able to show that the ID–OOD performance gap cannot be closed by merely drawing additional samples from the same training distribution.

#### Effect of training set diversity

Increasing training set diversity is widely held to be one of the most effective strategies for improving model generalizability and is frequently cited as future work in plant detection studies [6,30–32]. However, relatively few studies have conducted controlled experiments that assess the effects of training set diversity on model performance.

Using images of 3 kinds of broccoli cultivars, Blok et al. [33] showed that shared features from related domains could be mutually beneficial. They found that a model could learn to detect a particular cultivar with relatively few training examples, provided that it was also exposed to numerous examples of the other 2 cultivars.

Using 3 cotton seedling image sets, Jiang et al. [29] showed that OOD model performance depends on the relative diversities of the training and test sets. A model will generalize better when its training set is diverse enough to “cover” the diversity seen in the OOD test set.

In an experiment similar to our own, Ruigrok et al. [8] varied the number of domains from which they drew training samples while maintaining the same training set size. They found that models with more diverse training data generally performed better on OOD test sets. Additionally, they found that diverse models delivered a more consistent performance across training replications.

Our own experiment lends support to these findings, providing evidence that training set diversity improves model generalizability and consistency. On average, our models with diverse training sets showed stronger OOD performance than any of our non-diverse models. Additionally, our diverse models performed more consistently across replications than most of our non-diverse models.

#### Effect of training set quality

Previous work has shown that the performance and reliability of detection models are influenced by the quality of their training data [14,34]. However, creating pixel-precise bounding box annotations is time-consuming, and in certain applications, these lengthy annotation times cannot always be afforded. For example, if a model’s plant count estimates are needed to inform remedial field management decisions (such as determining if reseeding is necessary), it is essential that these counts are delivered in a timely manner.

For this reason, it is useful to study the effects of training on noisy annotations. Humans can create loose box annotations much more quickly than highly precise ones. Additionally, various plant detection studies have developed or employed automated techniques for generating low-quality bounding box annotations [35–37]. If these noisy annotations can be used to train effective detection models, large savings in human effort and time can be made.

Various studies have examined the effects of different kinds of annotation noise on model performance. Most existing studies examine the rate at which mean average precision (mAP) degrades as the noisiness of the training annotations increases. Agnew et al. [38] examined the effects of training on dilated bounding boxes from the COCO and Cityscapes datasets. They found that mAP degradation was variable across object classes. Xu et al. [39] removed box annotations from the COCO and Pascal VOC datasets, showing that several commonly used object detection architectures are highly sensitive to this type of noise.

Annotation noise studies that use agricultural object detection datasets are uncommon. Yuan et al. [40] explored how mAP degraded when annotations were removed from an apple bud dataset. Dong et al. [41] used a paprika plant dataset to experiment with 5 different types of annotation noise: missing boxes, incorrect class labels, box positional noise, box size noise, and annotation of blurry objects. They showed that certain types of noise, such as box positional noise, are more destructive to mAP performance than others.

While our own annotation quality experiments focused on only 2 types of annotation noise (box dilation and box removal), we explored how these types of annotation noise affect metrics other than mAP. By reporting different metrics, we showed that different types of noise result in different model behaviors. Depending on the application context, certain model behaviors may be more acceptable than others. For example, precise object counts are highly valued in aerial plant detection tasks. It is therefore critical that plant detection datasets are not missing significant numbers of bounding box annotations, as this type of annotation noise strongly affects the ability of detection models to generate accurate object counts. At the same time, we showed that models trained on dilated bounding boxes can still generate accurate object counts. Therefore, if count accuracy is the primary concern of the end-user, pixel-precise box annotations may not be required.

### Contributions and future work

Drone-based plant counting techniques have the potential to greatly improve the accuracy of early-season field health assessments. However, in order to supplant manual counting methods, plant detection models must be capable of delivering accurate results across the highly diverse conditions found in real-world environments.

In this paper, we investigated 3 training set properties that play an integral role in improving model generalizability: size, diversity, and quality. We showed that each of these aspects can strongly influence a model’s performance in unseen test conditions. We also demonstrated that the relative importance of these properties may vary, depending on the goals of the model user.

In addition to our experiments, we presented a large annotated dataset of early-season canola field imagery. We showed that our dataset can be used to train models that generalize well to newly acquired image sets of canola fields with similar spatial resolution.

Finally, we presented Canola Counter, a web tool for training and applying plant detection models. By enabling crop scientists to work directly with object detection models, Canola Counter can expedite the delivery of plant population estimates. The tool’s various outputs provide a rich characterization of stand establishment and overall field health.

Our dataset captures several real-world sources of distribution shift: changes in lighting conditions, growth stage variation, and differences in occlusion and plant clustering. Our experiments show that our training data provide good leverage in overcoming these sources of distribution shift in aerial canola seedling imagery. However, other sources of distribution shift that are commonly encountered in aerial plant detection contexts are not found within our dataset: sensor type variation, changes in camera height, and large geographic shifts. Expanding our dataset to include these factors of variation would be valuable future work.

In our experiments, we used random assignment to determine which image sets would be used for training and which image sets would be used for OOD testing. The use of a random split allowed us to realistically assess the quantity, diversity, and quality of training data that were needed to generalize to newly acquired image sets. However, a more controlled data split would have allowed us to explore the effects of a specific source of distribution shift. Assessing the distribution shifts caused by non-random data splits would be interesting future work. For example, our canola seedling images can be separated based on growth stage, severity of weed presence, or lighting conditions. Certain image set attributes will undoubtedly be more difficult to generalize across than others. Some data dimensions, such as lighting changes, may be effectively managed through data augmentation techniques. Other causes of distribution shift will be more challenging to overcome; developing effective methods for mitigating different sources of distribution shift would be highly valuable work. Since we have made our dataset publicly available, other researchers can use our dataset to perform these investigations.

Finally, in this study we examined the effects of training set size, diversity, and quality in isolation. However, in reality, it is the combination of these 3 properties that determines the utility of a dataset for training generalizable models. Examining the ways in which these 3 attributes interact with each other is therefore important future work.

For example, we conjecture that diverse training sets will have more potential for scalability as training set size increases. It would also be interesting to investigate trade-offs between training set diversity and annotation quality. Under what conditions will generalizability improve when a model’s training set is augmented by low-quality data? A better understanding of these and other questions will facilitate the development of plant detection models that are more performant and generalizable.

## Data Availability

The canola seedling dataset used in this study is publicly available and can be found at: https://doi.org/10.5281/zenodo.11055599. The Canola Counter tool is open source and is available at: https://github.com/eandvaag/agricounter.
